# Presentations, Causes and Outcomes of Drug-Induced Liver Injury in Egypt

**DOI:** 10.1038/s41598-020-61872-9

**Published:** 2020-03-20

**Authors:** Omkolthoum Alhaddad, Maha Elsabaawy, Eman Abdelsameea, Ayat Abdallah, Ahmed Shabaan, Nermine Ehsan, Ahmed Elrefaey, Dalia Elsabaawy, Mohsen Salama

**Affiliations:** 1 Hepatology and gastroenterology Department, National Liver Institute, Menoufia University, Shebin El-Kom, Egypt; 2 Epidemiology and Preventive Medicine Department, National Liver Institute, Menoufia University, Shebin El-Kom, Egypt; 3 Clinical Pharmacy, National Liver Institute, Menoufia University, Shebin El-Kom, Egypt; 4 Pathology Department, National Liver Institute, Menoufia University, Shebin El-Kom, Egypt

**Keywords:** Drug safety, Hepatitis

## Abstract

Drug-induced liver injury (DILI) is a frequent cause of liver injury and acute liver failure. We aimed to review all hospitalized DILI cases in a tertiary Egyptian center from January 2015 through January 2016. Cases with elevated alanine aminotransferase more than 3-fold and/or alkaline phosphatase more than 2-fold the upper limit of normal value were prospectively recruited and followed for one year. Drug history, liver biopsy whenever feasible and application of Roussel Uclaf Causality Assessment Method (RUCAM) were the diagnostic prerequisites after exclusion of other etiologies of acute liver injury. In order of frequency, the incriminated drugs were: Diclofenac (31 cases, 41.3%), amoxicillin-clavulanate (14 cases, 18.7%), halothane toxicity (8 cases, 10.7%), ibuprofen (4 cases, 5.3%), Khat (3 cases, 4%), tramadol (3 cases, 4%), Sofosbuvir with ribavirin (2 cases, 2.7%), and acetylsalicylic acid (2 cases, 2.7%) with one offending drug in 93.3% of cases. Forty-four cases (58.7%) were males; while 56 cases (74.7%) had HCV related chronic liver disease. Thirty-two cases (42.7%) presented with pattern of hepatocellular injury, while 23 cases (30.7%) were with cholestasis, and 20 cases (20.7%) with a mixed hepatocellular/cholestatic injury. One case received a transplant (0.75%), 7 cases died (9.3%), 23 cases (30.6%) developed liver decompensation (hepatic encephalopathy and ascites), and 44 cases completely resolved (58.7%). In conclusion, Diclofenac is the commonest offender in DILI occurrence in an Egyptian cohort. Age and prothrombin concentration were the only predictors of unfavorable outcomes of DILI.

## Introduction

Drug induced liver injury (DILI) is one of the least understood areas in hepatology research. Since 1960, DILI is considered one of the commonest causes of withdrawal of approved medications from the marketplace^1^. The mounting rates of DILI reports had notified it as a leading confusion of acute liver failure (ALF)^2^. Despite critical DILI presentations, resolution of injury is common. Typical incriminating factors that were attributed to DILI occurrence were the medical personnel’s’ unawareness of the drugs morbid effects upon the liver and the availability of the over the counter drugs to the general public. On review of these cases, the main challenges that presented were the paucity of national well-structured notification programs with a subsequent substantial shortage of data registries. In addition, the lack of an objective test for the diagnosis of DILI, similarities to other liver diseases and the difficulty in defining the offending drug in patients on many medicines, complicated its recognition^3^. Nevertheless, a high index of suspicion is necessarily to establish the diagnosis^4^. In Egypt, there is lack in epidemiological data and DILI registry. This was the reason for designing this prospective study investigating DILI occurrence, demographic, clinical, laboratory and histopathological characteristics along with disease outcomes in an Egyptian cohort.

## Patients and methods

This prospective study was conducted on all patients diagnosed with DILI in hepatology and gastroenterology department, National Liver Institute (NLI), Menoufia University in the period from January 2015 through January 2016. Patients were followed up to one year after diagnosis. Patients’ written informed consent and approval of the ethical committee of NLI (The institution Review Board of NLI, Menoufia University) were prerequisites for inclusion in this study. All methods were performed in accordance with the relevant guidelines and regulations.

As defined by Aithal *et al*.^5^, DILI was defined by isolated increase of ALT ≥ 5 × upper limit of normal value (ULN) or increase of ALT values ≥ 3 × ULN and concomitant increase of total bilirubin (TB) values ≥ 2 × ULN or increase of alkaline phosphatase (ALP) values ≥ 2 × ULN and concomitant increase of gamma-glutamyl-transferase (γ-GT).

Acute on chronic liver failure (ACLF) was defined as acute hepatic injury manifested as jaundice (with bilirubin ≥5 mg/dl), coagulopathy (with international normalized ratio ≥1.5 or prothrombin activity <40%), and complicated within 4 weeks by ascites and/or hepatic encephalopathy with previously diagnosed or undiagnosed chronic liver disease (CLD)^6^. Evident drug history, along with exclusion of other causes of acute liver injury in patients with HCV related CLD were the criteria of inclusion in this study. No cases with hepatitis B Virus (HBV) monoinfection or co-infection with HCV were included in this study.

All cases were considered as suggestive of DILI according to meticulous drug history and causality assessed using Roussel Uclaf Causality Assessment Method (RUCAM) score^7^. Only cases that achieved values denoting highly probable or probable were included in the study.

The pattern of DILI was defined according to the R value which is defined as the ratio of ALT to ALP as multiples of their upper normal limits. Hepatocellular injury is defined when R value equals more than 5, mixed injury if it is between 2 and 5, while it is cholestatic injury when R value equals less than 2^5^.

Eosinophilia was defined as absolute eosinophil counts exceeding 450 to 550 cells/µL, depending on laboratory standards^8^.

Immune mediated DILI was defined by presence of features of type IV hypersensitivity reactions (fatigue, high-grade fever, arthralgia, skin rash, facial edema, and generalized itching)^9^.

Complete recovery was defined as complete restoration of the basal clinical and biochemical profile within 6 months of injury. Poor (unfavorable) outcome is defined as failure of restoration of the clinical and biochemical profile up to 6 months. Death, urgent liver transplantation, multiorgan failure, decompensation of compensated liver disease, and drug induced chronic liver injury in acute cases all were considered unfavorable DILI outcomes according to Lianos *et al*.^10^. Chronicity was defined as abnormal liver biochemistry, imaging test or histology one year after DILI recognition according to Medina-Caliz *et al*.^11^.

All patients were subjected to the following:

1. A detailed history taking especially drug history (time, dose, type, duration and history of same or similar drug intake)
2. Physical examination.
3. Biochemical tests: liver function tests, kidney function tests, and complete blood count
4. Serological tests:
  All other causes of acute liver injury were excluded either:
  Viral: hepatitis B surface antigen (HBsAg), anti-hepatitis B core (HBc)-IgG, anti-HBc-IgM, anti-hepatitis E virus (HEV IgM), anti-Epstein–Barr virus (EBV)-IgM, anti-Cytomegalovirus (CMV) -IgM, Hepatitis C virus antibodies (HCV Abs) and quantitative HCV RNA by PCR. Anti-hepatitis A virus Ab, Anti- human immunodeficiency virus (HIV)-Ab and anti- herpes simplex virus (HSV) -IgM. Autoimmune: Anti-nuclear antibodies (ANA), Anti-smooth muscle antibodies (ASMA), Anti-mitochondrial antibodies (AMA), and Anti- Liver-Kidney-Microsome (LKM)-1 antibodies. Metabolic: Serum iron, total iron binding capacity, serum ceruloplasmin, 24 hours urinary copper excretion.
5. Imaging: Abdominal ultrasonography and computed tomography (CT) if needed.
6. Liver biopsy: was performed for patients who were eligible (with international normalized ratio <1.5 or prothrombin concentration ≥40%). The reported pathological features suggestive of DILI included: mild inflammatory changes in portal areas compared with those in liver parenchyma, demarcated perivenular (acinar zone 3) necrosis, confluent necrosis, cholestasis, minimal hepatitis with canalicular cholestasis, fat deposits in hepatocytes (≥30%), abundant neutrophil and or eosinophil infiltration, presence of epithelioid-cell granulomas, biliary damage and inflammation, and severe cholestasis^12^.

### Statistical analysis

The data analyzed using SPSS (Statistical Package for Social Science) program for statistical analysis, (version 20; Inc., Chicago. IL). Two types of statistics were done; 1. Descriptive statistics: where quantitative data have been shown as mean, and SD, while qualitative data was expressed as frequency and percent, 2. Analytical statistics: where Chi- square test has been used to measure the association between qualitative variables, and Mann- Whitney test has been used to compare differences between two independent groups regarding variables with quantitative not normally distributed data. Fisher exact test was used for 2 × 2 qualitative variables when more than 25% of the cells have expected count less than 5. The p-value was considered statistically significant when it was <0.05.

## Results

Seventy-five DILI cases were enrolled and followed for up to 6 months. Nineteen cases presented with acute DILI, while the remaining 56 cases presented with acute DILI on top of chronic HCV infection.

### Comparison between acute DILI and acute DILI on top of chronic HCV infection revealed

There was statistically significant difference between the two groups regarding gender with male predominance in acute DILI on top of chronic HCV and female predominance in acute DILI (p = 0.025) **(**Table 1**)**. Regarding age: The group of acute on top of chronic HCV were significantly older (46.4 ± 12.3 years old) (p = 0.001) **(**Table 1**)**. Laboratory findings at presentation had statistically significant difference between the two groups as regards aspartate aminotransferase (AST), alanine aminotransferase (ALT), prothrombin concentration and platelet counts **(**Table 1**)**. Regarding R value; in acute DILI, the injury was mostly hepatocellular with an R value >5 in 89.5% of cases, with only 2 cases (10.5%) presented by mixed hepatocellular/cholestatic injury with an R ratio between 2 and 5. While in cases of acute on top of chronic HCV infection: the injury was cholestatic with an R ratio <2 in 41.1%, mixed hepatocellular/cholestatic type with an R ratio between 2 and 5 in 32.1% with the hepatocellular injury with an R ratio >5 in 26.8% as the least common significantly different (p < 0.001) **(**Table 1**)**. The eosinophilia presentation was evident in 31.6% of acute DILI cases while present in only 7.1% in cases of acute on top of chronic hepatitis C, with a statistical significance (p = 0.013) **(**Table 1**)**.

**Table 1:** Data of cases at presentation.

Studied variables	Acute n = 19		Acute on top of Chronic n = 56		P-value
	n	%	n	%	
**Gender:**					
Male	7	36.8	37	66.1	0.025
Female	12	63.2	19	33.9	
**Symptoms:**					
Jaundice + abdominal pain	2	10.5	15	26.8	0.192
Jaundice + abdominal pain + itching	3	15.8	3	5.4	
Jaundice	11	57.9	34	60.7	
Jaundice + itching	3	15.8	4	7.1	
**Eosinophilia**	6	31.6	4	7.1	0.013*
**Immune Mediated:**	9	47.4	12	21.4	0.030
**R value:**					
Less than 2	0	0.0	23	41.1	<0.001
From 2–5	2	10.5	18	32.1	
More than 5	17	89.5	15	26.8	
RUCAM score**:					
**Probable total**	**1**	**0.0**	**25**	**44.6**	0.044
6	0	0.0	2	3.6	
7	0	0.0	3	5.5	
8	1	5.5	20	36.4	
**Highly probable total**	**17**	**89.5**	**30**	**53.6**	
9	7	38.9	16	29.1	
10	7	38.9	12	21.8	
11	3	16.7	2	3.6	
**Age: (years)**	**Mean ± SD** **33.6 ± 11.8**		**Mean ± SD** **46.4 ± 12.3**		0.001*
AST * Iu U/L	806.7 ± 602.7		445.5 ± 688.9		<0.001
ALT * Iu U/L	1028.7 ± 667.2		277.7 ± 704.8		<0.001**
**ALP Iu U/L**	177.4 ± 61.9		188.9 ± 114.3		0.67**
PC * %	67.2 ± 21.0		53.8 ± 22.5		0.024**
**Total bilirubin mg/dl**	17.41 ± 1.3		20.2 ± 8.2		0.26**
**Direct bilirubin mg/dl**	13.9 ± 9.2		16.4 ± 6.8		0.289**
**Heamoglobin g/dl**	13.3 ± 1.3		12.4 ± 1.8		0.054
**Platelet count 10** ^**3**^**/dl**	258.5 ± 72.7		181 ± 96.4		0.001**
WBCs * 10^**3**^**/dl**	24.9 ± 78.7		12.3 ± 34.8		0.99**

*p-value value of significance. SD = standard deviation. n = number, **% =** percentage, AST = Aspartate Aminotransferase, ALT = Alanine Aminotransferase, ALP = Alkaline phosphatase, P.C = prothrombin concentration, WBCs = White Blood Cells, **Two cases missing in RUCAM score, one in each group. RUCAM **=** Roussel Uclaf Causality Assessment Method.

### Drugs incriminated in DILI occurrence

Diclofenac was the most common offending agent in this study responsible in 31 (41.3%) patients, amoxicillin clavulanate was the second cause (18.6%), halothane was the third (10.7%), followed by ibuprofen which was the causative drugs in 4 cases (5.3%), khat and tramadol were the causative drugs in 3 cases (4.0%) for each and sofosbuvir plus ribavirin dual therapy was the causative drugs in 2 (2.7%) cases, the same prevalence for acetyl salicylic acid and chemotherapeutics (indoxan and vincristine) induced DILI (2.7%). Other drugs incriminated with single occurrence in our study (1.3%) were carbimazole, tenoxicam, methimazoles, progesterone therapy, carbamazepine and treatment with triple therapy by sofosbuvir plus interferon and ribavirin **(**Table 2**)**. Liver injury associated with amoxicillin clavulanic acid was cholestatic in 57.1% and mixed in 42.9% of cases. The mean age of cases presenting with mixed versus cholestatic liver injury in those due to amoxicillin- clavulanic acid were 31.1, 48.6 years respectively. Halothane was mainly hepatocellular in 75% of cases, and 25% for both cholestatic and mixed forms. Diclofenac injury was hepatocellular in 54.8% and equally cholestatic or mixed in the remaining cases. Ibuprofen cases mainly presented with hepatocellular injury (75.0% of cases). All 3 cases of khat induced liver injury were hepatocellular (Table 2). Time interval between drug intake and symptoms was variable; it was a short interval with halothane, diclofenac and amoxicillin clavulanic acid induced liver injury, with mean ± SD 8.3 ± 3.3, 11.2 ± 5.3, 3.9 ± 1.5 days respectively. Longer intervals were shown with tramadol and khat induced liver injury with mean ± SD 29.6 ± 12.6 and 25.6 ± 4.1 days respectively; with more than 2 months interval in carbamazepine and carbimazole induced liver injury (Table 2).

**Table 2:** Drugs incriminated in DILI occurrence.

	Total (n = 75) N (%)	Time interval (days) M ± SD	cholestatic N (%)	Mixed N (%)	Hepatocellular N (%)	Acute N (%)	Chronic N (%)
Diclofenac	31 (41.3)	11.2 ± 5.3	7(22.6)	7(22.6)	17(54.8)	10 (53)	21.(37.5)
Amoxicillin Clavulanic acid	14 (18.6)	3.9 ± 1.5	8 (57.1)	6(42.9)	0	5(26.5)	9(16.1)
Halothane	8 (10.7)	8.3 ± 3.3	0	2(25)	6 (75)	5(26.5)	3(5.4)
Interferon, sofosbuvir and ribavirin	1 (1.3)	45 ± 0	0	1(100)	0	0	1(1.8)
Chemotherapeutics	2 (2.7)	60 ± 0	2 (100)	0	0	1(5.3)	1(1.8)
Acetyl salicylic acid	2 (2.7)	12 ± 2	0	1(50)	1(50)	0	2(3.6)
Khat	3 (4)	25.6 ± 4.1	0	0	3(100)	1(5.3)	2(3.6)
Tramadol	3 (4)	29.6 ± 12.6	1 (33.3)	2(66.7)	0	1(5.3)	2(3.6)
Ibuprofen	4 (5.3)	8.5 ± 2.29	1 (25)	0	3(75)	2(10.5)	2(3.6)
Sofosbuvir ribavirin	2 (2.7)	67.5 ± 7.5	2 (100)	0		0	2(3.6)
Carbimazole	1 (1.3)	60 ± 0	0	0	1(100)	1(5.3)	0
Tenoxicam	1 (1.3)	6 ± 0	0	0	1(100)	1(5.3)	0
Methimazole	1 (1.3)	7 ± 0	1 (100)	0	0	1(5.3)	0
Progesterone	1 (1.3)	21 ± 0	0	1 (100)	0	0	1(1.8)
Carbamazepine	1 (1.3)	60 ± 0	1 (100)	0	0	1(5.3)	0

*p-value value of significance. n = number, **% =** percentage, M = Mean, SD = standard deviation.

### Outcomes

Complete recovery occurred in 58.7% of cases. Those with unfavorable outcome included 9.3% of cases who died during follow up, 1.3% of them were transferred to urgent liver transplantation and 30.7% of patients had persistent liver injury for more than 6 months. The only patient who was transferred to transplantation due to DILI was 30 years old female who presented with acute hepatocellular injury due to halothane.

**Figure 1:**
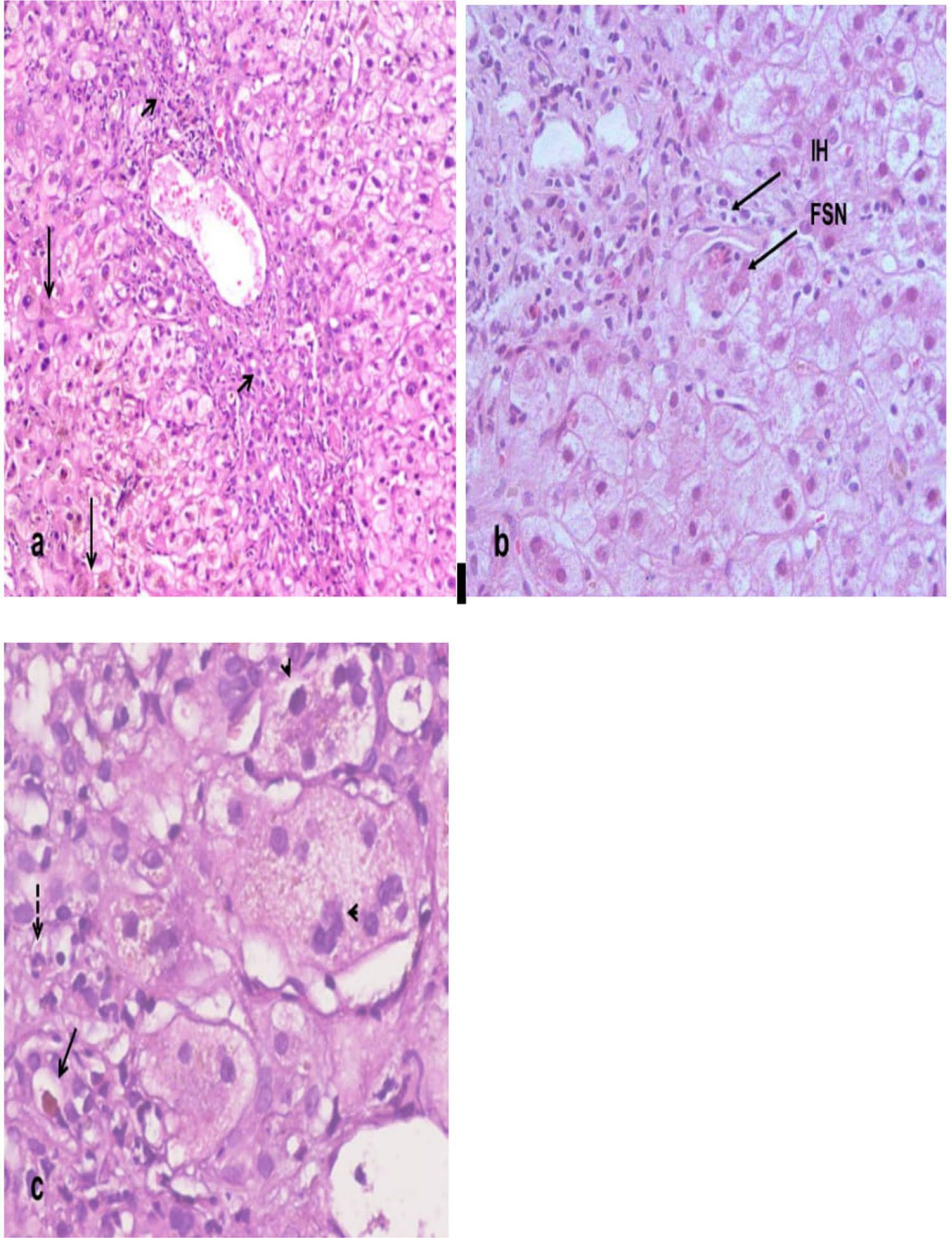
Histopathological types of liver injury in DILI. (**a**) Cholestatic liver injury showing mixed inflammatory cellular infiltrate (short arrows) in portal tracts attaching bile ducts, ductular proliferation and cholestasis in cytoplasm of hepatocytes (long arrows), Original mag. × 100. (**b**) Hepatocellular liver injury showing portal tact inflammation, interface hepatitis (IH) and mild hepatitic changes in the form of focal spotty necrosis (FSN) of hepatocytes, Original mag. × 200. (**c**) Mixed hepatocellular/cholestatic injury showing giant cell transformation of hepatocytes (arrow heads), neutrophilic infiltration (dashed arrow) and cholestatic rosettes (arrow), Original magnification × 200.

There were 13 cases that underwent liver biopsies, in 6 cases focal necrosis with lymphocytes and plasma cells caused by diclofenac in 5 cases and amoxicillin clavulanic acid in 1 case, all of which completely recovered. Auto immune with abundant eosinophilia was founded in 2 khat and 1 diclofenac cases. In two cases with amoxicillin clavulanic DILI: one presented with severe cholestasis, while steatohepatitis was prevalent in the other one. Cholestatic hepatitis was found in one diclofenac case, while centrilobular necrosis was found in one halothane induced case (Table 3 and Fig. 1**)**.

**Table 3:** Descriptive data of DILI cases who underwent liver biopsy.

	Steatohepatitis (n = 1)		Centrilobular necrosis (n = 1)		Cholestatic hepatitis (n = 1)		Severe cholestasis (n = 1)		Auto immune with abundant eosinophilia (n = 3)		focal necrosis with lymphocytes and plasma cells (n = 6)	
	N	%	N	%	N	%	N	%	N	%	N	%
**Drugs**												
Amoxicillin clavulinic	1	100.0	0	0	0	0	1	100	0	0.00	1	16.7
Khat	0	0	0	0	0	0	0	0	2	66.7	0	0
Diclofenac	0	0	0	0	1	100	0	0	1	33.3	5	83.3
Halothane	0	0	1	100	0	0	0	0	0	0	0	0
**Outcome**												
Complete recovery	1	100	0	0	1	100	1	100	2	66.7	6	100
Poor outcome	0	0	1	100	0	0	0	0	1	33.3	0	0

N** =** number, % percentage.

There were seven deceased cases; the mean age was 58.6 ± 3.2 years old, 5 of them were males (71.4%), one (14.3%) presented with hepatocellular injury, 5 (71.4%) with cholestatic injury and one (14.3%) presented by mixed injury, all having chronic liver disease. Four cases were from diclofenac induced liver injury, two patients with chemotherapy (indoxan, vincristine) induced liver injury and one case due to sofosbuvir plus ribavirin induced liver injury (Table 4).

**Table 4:** Descriptive data of the seven deceased cases.

Age		Mean + SD 58.57 + 3.2 years	
		N	%
Gender	Male	5	71.4
	Female	2	28.6
Type of liver injury	Hepatocellular	1	14.3
	Cholestatic	5	71.4
	Mixed	1	14.3
Presentation	Acute	0	0
	Acute on top of chronic	7	100
Causative drug	Diclofenac	4	57.1
	Chemotherapy(indoxan, vincristine)	2	28.6
	Sofosbuvir &ribavirin	1	14.3

Univariate analysis of factors associated with the outcome revealed the following: there was statistically significant relation between younger age and good outcomes, (p < 0.001) and eosinophilia was present more often in patients who completely recovered (20.5%). Higher prothrombin concentration was associated with good outcome. There was statistically significant relation between higher serum bilirubin at presentation and poor outcome (p = 0.027) **(**Table 5**)**.

**Table 5:** Univariate analysis of factors affecting outcome.

Studied variables	Complete Recovery (n = 44)		Poor outcome (n = 31)		p-value
	n	%	n	%	
**Gender:**					**0.929**
Male	26	59.1	18	58.1	
Female	18	40.9	13	41.9	
**Age (years)**	**Mean ± SD** **37.3 ± 12.5**		**Mean ± SD** **51.3 ± 9.8**		<0.001*
**Presentation:**	18	40.9	1	3.2	<0.001*
▪ Acute	26	59.1	30	96.8	
▪ Acute on top of chronic					
**Type of liver injury:**					0.004
▪ Cholestatic	7	15.9	16	51.6	
▪ Hepatocellular	23	52.3	9	29	
▪ Mixed	14	31.8	6	19.4	
**Metabolism:**	40	90.9	28	90.3	0.931*
▪ Hepatic	4	9.1	3	9.7	
▪ Renal					
**Immune mediated:**	16	36.4	5	16.1	0.055**
**Eosinophilia**	9	20.5	1	3.2	0.039**
**AST (U/L)**	**Mean ± SD 612.8 ± 741.4**		**Mean ± SD 429.4 ± 584.2**		0.089*
**ALT(U/L)**	Mean ± SD 602.5 ± 670.4		Mean ± SD 277.06 ± 360.84		0.007
**Prothrombin Concentration %**	66.06 ± 19.15		44.82 ± 21.96		<0.001
**Alkaline phosphatase (U/L)**	198.20 ± 116.97		168.74 ± 78.60		0.187
**Bilirubin total mg/dl**	17.53 ± 9.47		22.35 ± 8.03		0.027
**Bilirubin direct mg/dl**	14.27 ± 7.85		17.90 ± 6.65		0.04
RUCAM score***					0.209
**Probable total**	**14**	**31.8**	**12**	**38.7**	
▪ 6	0	0.0	2	6.9	
▪ 7	2	4.5	1	3.5	
▪ 8	12	27.3	9	31.0	
**Highly probable total**	**30**	**68.2**	**19**	**61.3**	
▪ 9	15	34.1	8	27.6	
▪ 10	10	22.7	9	31.0	
▪ 11	5	11.4	2	6.9	
**Causative agent:**					0.304
⦁ Amoxicillin clavulanic acid	9	20.4	5	16.1	
⦁ Halothane	5	11.4	3	9.7	
⦁ Diclofenac	20	45.5	11	35.5	
⦁ Interferon &sofosbuvir& ribavirin	1	2.3	0	0	
⦁ Chemotherapy (indoxan, vincristine)	0	0	2	6.5	
⦁ Acetyl salicylic acid	0	0	2	6.5	
⦁ Khat	2	4.5	1	3.2	
⦁ Tramadol	2	4.5	1	3.2	
⦁ Ibuprofen	3	6.8	1	3.2	
⦁ sofosbuvir ribavirin	0	0	2	6.5	
⦁ Carbimazole	1	2.3	0	0.0	
⦁ Tenoxicam	1	2.3	0	0.0	
⦁ Metamizole	0	0	1	3.2	
⦁ Hormonal therapy (progesterone)	0	0	1	3.2	
⦁ Carbamazepine	0	0	1	3.2	

*p-value statistically significant. n = number, % = percentage. n = number, SD = standard deviation. AST = Aspartate Aminotransferase, ALT = Alanine Aminotransferase, n = number, SD = standard deviation, RUCAM = Roussel Uclaf Causality Assessment Method, % = percentage, *Two cases are missing in RUCAM score of bad outcome group of patients (n = 29).

Multivariate analysis for detection of predictors of complete recovery among studied patients revealed that age less than 37.4 ± 12.5 years (p-value < 0.001 with odds ratio 0.898) along with higher prothrombin concentrations more than 66.1 ± 19.2% (p-value = 0.001 with odds ratio 1.06) were the only predictors of good outcome (Table 6).

**Table 6:** Multivariate analysis for detection of predictors of complete recovery.

Variables	p-value	OR*	95% CI	
			Lower	Upper
**Age years** (less than **37.4** ± **12.5 years)**	<0.001	0.898	0.852	0.948
**Prothrombin concentration** (more than 66.1 ± 19. 2%)	0.001	1.06	1.026	1.096

*OR (odds ratio), CI; confidence interval.

## Discussion

As most DILI studies are currently available from well developed nations, like Iceland, Spain and the United States, it is an important to characterize the features of DILI in a developing country. In a French study from a northern city, they reported an incidence of approximately 14 per 100,000 DILI patients per year^13^. A Korean study found a rate of 12/100,000 persons/year^14^. Recent study from Iceland estimated an annual incidence rate of DILI of 19.1 cases per 100,000 inhabitants^15^.

Many prospective data registry studies (the American and Spanish studies), failed to report the relative incidence of DILI^16,17^.

In our study, similarly the true DILI incidence estimates could not accurately be determined. Due to: 1) possibility of undiagnosed cases along with those managed in outpatient clinics 2) those taken care of private health sector, as usual as many other referral centers. Consequently, we believed that our results are most likely underestimation of the true DILI occurrence in Egypt.

DILI prevalence in NLI accounted for 1.38% of all admissions over one year (75 patients from 5452 cases admitted to the Hepatology department from November 2014 to November 2015).

Similar to our study, Devarbhavi *et al*. had reported DILI contributed to 1.4% of all gastrointestinal admissions and 2.5% of hepatobiliary admissions over one year^18^. In a Thailand study; 1.2% of admissions was due to DILI^19^. The results of Meier *et al*., 2005 were with 1.4% DILI occurrence in hospitalized patients in Switzerland^20^. Also similar to our results Idilman *et al*. had estimated 3.1% prevalence of DILI in patients admitted to a Turkish academic medical center from January 2001 to June 2007^21^.

The higher prevalence of hyperthyroidism and tuberculosis in China had alleged their therapies to be the second and third most reported hepatotoxic drugs^22^. Licata *et al*. reported that antibiotics were involved for 23.4%, NSAIDs for 35.5%, immunosuppressants for 10.9%, statins for 4.3%, anti-platelets and anti-psychiatric drugs for 7.6%^23^.

In the current study, Diclofenac was the drug leading cause of DILI in this Egyptian cohort (41.3%). Amoxicillin clavulanate was the second most common (18.6%), while Halothane was the third in the list (10.7%).

The higher Egyptian rates of Cesarean section (CS) labors compared to the international reviews might explain the rise in halothane DILI reported in Egyptian females^24^.

In the present study, age appeared to be a risk factor for DILI occurrence with the highest rates in fifties. This might reflect the cumulative risk of drug exposure over years, and or the susceptibility of consumption of drug metabolizing and excreting hepatic enzymes. These results were consistent with many studies identifying age as a risk factor for DILI occurrence^25^.

Contradictory were the results of the large Spanish study based on hepatotoxicity registries with more than 600 patients; where age risk was not substantiated^26^.

Regarding outcomes in this study, 68 patients (91.7%) of our cohorts were survivors, one of them survived following liver transplantation. Complete recovery with return to normal liver functions in acute DILI or to pre-injury status in acute on top of chronic HCV cases was reported in 58.7% of cases. Poor outcome was experienced in 41.3% of cases with persistently deranged liver function tests for more than 6 months. Consequently, one patient (1.3%) had developed acute liver failure requiring urgent liver transplantation and 7 patients (9.3%) died. In concordance, Ostapowicz *et al*. had mentioned 8% mortality with 2% requiring urgent liver transplantation^27^. Examining drug related outcomes in 64.3% (9 patients out of 14) with amoxicillin clavulanic acid DILI, 62.5% with halothane DILI (5 patients out of 8) and 64.5% (20 patients out of 31 patients) of those with diclofenac related DILI. Khat, tramadol and ibuprofen were also associated with complete recovery while acetylsalicylic acid and chemotherapy with cyclophosphamide and vincristine induced DILI were associated with poor outcome with incomplete recovery to normal. Those drug postulations lacked similarities in literature for the small number of DILI cases caused by these agents. Björnsson and Olsson in 2005 reported that mortality reached 40% with halothane-induced liver injury, whereas all patients with erythromycin-induced liver injury survived^28^. In another study by Björnsson *et al*. revealed that only 1 out of 35 (3%) cases of erythromycin-induced liver injury were associated with fatal outcome^29^.

In our study, patients with amoxicillin clavulanic acid induced DILI completely recovered from their injury. Mortality in diclofenac induced DILI cases was 12.9% with complete recovery in 64.5%. While the two cases who presented with cholestasis and received chemotherapy (cyclophosphamide and vincristine) had abruptly died. Halothane induced DILI represented 62.5% of cases and they had complete recovery. All khat induced DILI cases presented with hepatocellular injury (3 cases) with complete recovery in 66.7% of khat cases.

The prognosis of patients with cholestatic DILI was mostly presumed to be worse than both hepatocellular and mixed liver injury patterns^6^. According to our cohort, cholestatic injuries represented more than half of poor outcome cases.

Björnsson *et al*. reported a worse prognosis in patients with acute hepatocellular DILI than those with cholestatic or mixed liver injury pattern^29^. They had reported that in the first study in 2007 hepatocellular injury patterns are more common in younger patients and cholestatic patterns in older patients.

However, in the Ibáñez *et al*. study the type of injury was an independent element for outcome determination^30^.

Regarding the presence of peripheral eosinophilia, results from this study revealed statistically significant associated with favorable outcomes. It was found in 20.5% of cases who were completely recovered and in only 3.2% of cases with poor outcome. Similarly, eosinophilia was reported in 49 of the 185 (26.5%) DILI patients in another study but was not present in any of the 12 patients who died or underwent liver transplant surgery^31^. In the DILIN prospective study, no link between initial eosinophilic counts and outcome was documented^32^.

Regarding recovery, in our study, the median duration for normalization of liver function tests (LFTs) was 66 days. About 30.7% of patients reported persistent LFTs abnormalities for more than 6 months of DILI diagnosis.

This was consistent with De Valle *et al*., 2006, who showed a median duration for normalization of LFTs 64 days^33^. While, Ostapowicz *et al*., 2002, had showed 14% of his studied cohort suffered persistent LFTs derangements for more than 6 months^27^. More recently, Medina-Caliz *et al*.^11^ had nominated one year as the best end point to define chronic DILI. They reported that older age, dyslipidemia and severity of the acute episode are risk factors of chronic DILI.

Multivariate analysis performed in this cohort group, proved younger age along with higher prothrombin concentrations to be the only predictors of good DILI outcomes.

Conclusively, the failure in identifying the true DILI incidence in our study had yielded an abrupt urge of assembling an adapted national DILI notification network with more large-scale population-based DILI studies. Stratification of DILI national studies must shed more lights on the natural history of this disorder especially in country like Egypt cursed with the highest burden of HCV representing a gross risk component of the dynamics of DILI morbid outcomes.
